# Blending an implementation science framework with principles of proportionate universalism to support physical activity promotion in primary healthcare while addressing health inequities

**DOI:** 10.1186/s12961-020-00672-z

**Published:** 2021-01-18

**Authors:** Bojana Klepac Pogrmilovic, Sarah Linke, Melinda Craike

**Affiliations:** 1 Mitchell Institute for Education and Health Policy, Victoria University, Melbourne, Australia; 2 Family Medicine and Public Health, University of California, San Diego, USA; 3 Institute for Health and Sport (IHES), Victoria University, PO Box 14428, Melbourne, VIC 8001 Australia

**Keywords:** Physical activity promotion, Implementation, Policy, Practice, Primary healthcare, Health inequities, Proportionate universalism

## Abstract

Globally, insufficient physical activity (PA) is one of the main risk factors for premature mortality. Although insufficient PA is prevalent in nearly every demographic, people with socio-economic disadvantage participate in lower levels of PA than those who are more affluent, and this contributes to widening health inequities. PA promotion interventions in primary healthcare are effective and cost effective, however they are not widely implemented in practice. Further, current approaches that adopt a ‘universal’ approach to PA promotion do not consider or address the additional barriers experienced by people who experience socioeconomic disadvantages. To address the research to policy and practice gap, and taking Australia as a case study, this commentary proposes a novel model which blends an implementation science framework with the principles of proportionate universalism. Proportionate universalism is a principle suggesting that health interventions and policies need to be universal, not targeted, but with intensity and scale proportionate to the level of social need and/or disadvantage. Within this model, we propose interrelated and multi-level evidence-based policies and strategies to support PA promotion in primary healthcare while addressing health inequities. The principles outlined in the new model which blends proportionate (Pro) universalism principles and Practical, Robust Implementation and Sustainability Model (PRISM), ‘ProPRISM’ can be applied to the implementation of PA promotion interventions in health care settings in other high-income countries. Future studies should test the model and provide evidence of its effectiveness in improving implementation and patient health outcomes and cost-effectiveness. There is potential to expand the proposed model to other health sectors (e.g., secondary and tertiary care) and to address other chronic disease risk factors such as unhealthy diet, smoking, and alcohol consumption. Therefore, this approach has the potential to transform the delivery of health care to a prevention-focused health service model, which could reduce the prevalence and burden of chronic disease and health care costs in high-income countries.

## Introduction

Insufficient physical activity (PA) is identified by the World Health Organization (WHO) as one of the leading risk factors for global mortality [1] and has significant health and economic impacts on public health systems [2]. Australian modelling suggests that reducing the prevalence of physical inactivity by 10% will result in 6000 fewer incidents of disease, 2000 fewer deaths, 25,000 fewer Disability-adjusted life years, and reduce health care costs by $96 million per year [3]. When considering the national guidelines for both aerobic activity and strength training, only 15% of Australian adults achieve the weekly recommended levels of PA [4].

Physical activity is a complex behaviour, that is influenced by interrelated factors at the environmental, cultural, social, and individual levels of the Social Ecological Model. Social determinants of health, including socio-economic position (e.g., income and education) affect physical activity levels for multiple reasons, including less time to spend on physical activity if working multiple jobs to make ends meet or less access to safe places to be active. Of concern, people who experience socio-economic disadvantage participate in lower levels of PA than those who are more affluent, which contributes to the widening of health inequities in Australia [4].

Any attempts to increase PA need to address inequities in participation through consideration of the additional barriers experienced by people who are disadvantaged. These barriers include cost of living pressures; time pressures caused by work, family and carer duties; concerns about safety; poor quality and limited availability of facilities and spaces [5, 6]. There is also evidence of lower levels of service provision to support participation in PA in disadvantaged areas. For example, compared to more affluent areas, disadvantaged areas have a lower per capita proportion of exercise practitioners, with higher caseloads. This means that people living in areas of greater socio-economic disadvantage may have longer waiting times to access exercise practitioners and reduced length of consultation times [7], leading potentially to poorer health outcomes.

Investment in PA promotion has been identified as a ‘best buy’ for decision makers [1] and health care settings are recognised as important settings for PA promotion. PA promotion interventions in primary healthcare are effective at increasing PA [8], cost-effective [9], and supported by the WHO [10]. The most robust evidence supports a model of PA promotion in primary healthcare that includes routine screening, brief advice, and referral to an appropriately trained practitioner to deliver PA counselling. Evidence supports that five sessions can effectively increase PA [11] and that counselling sessions should be based on evidence-based strategies such as: goal setting and monitoring; supporting patient’s autonomy and preferences [12]; utilising the 5-A model (assess, advise, agree, assist, arrange) of counselling and behaviour change [13], together with a multi-sectoral approach that includes strategies to connect patients with local PA opportunities [12]. A multi-sectoral approach such as this progresses primary care-public health partnerships, which are increasingly recognised as crucial to supporting patient-centred care [14] and integrates healthcare services with other sectors to form place-based health systems that influence wider social, community, and economic drivers of health [10, 15]. This approach is also likely to be sustainable as it maximises the utilisation of existing resources in the community.

Importantly for reducing health inequities, PA promotion interventions in primary healthcare are one of the few interventions whose effectiveness has been demonstrated across diverse patient groups, including those experiencing socio-economic disadvantage [7]. Because of its effectiveness and potential for wide population reach, delivery of PA promotion interventions in routine primary healthcare could help to tackle high levels of inactivity and improve health systems, subsequently leading to substantial clinical, population health, and economic benefits [7–10]. However, it is important to note that even though PA interventions in primary healthcare are shown to be effective, it is only one setting where PA promotion should take place. To tackle the ‘pandemic of physical inactivity’ a whole system approach is necessary, which includes the development and implementation of effective government actions and policies on national and local levels, cross-sectoral collaboration between various sectors within the government (e.g. transport, health, education) as well as collaboration with non-government organisations, community, industry, and private sector [10].

Proportionate universalism suggests that health interventions (including policy) need to be universal, not targeted, but with intensity and scale proportionate to the level of disadvantage and/or social need [16]. Some evidence shows this approach has been successful in reducing health inequities [17, 18]. One study, “a concrete example of proportionate universalism” reported on effectiveness of a postnatal home visiting program in a disadvantaged area in Stockholm, Sweden [15]. A quasi-experimental study of United Kingdom neighbourhood renewal program also applied proportionate universalism in practice [16]. Although proportionate universalism has been recommended and is an increasingly popular principle in public health, there has been little guidance on how to operationalise this approach in policy and practice and how it may be integrated within existing frameworks to reduce health inequities [16]. Moreover, there is little to no evidence that this approach has been attempted in PA interventions, policy, or practice in Australia [19].

Evidence-based PA promotion interventions are rarely implemented and the research-to-practice and policy gap has been identified as a public health priority [20]. To improve the implementation of evidence-based PA promotion interventions in primary healthcare, evidence-based policies and strategies at multiple levels are needed to encourage uptake, implementation, and sustainability in practice. Taking Australia as a case study, in this commentary, we propose a novel model that blends an implementation science framework with the principles of proportionate universalism to support PA promotion in primary healthcare and reduce health inequities. The model can be used to inform policy and applied in health care settings in other high-income countries as the issue is internationally relevant and the model is flexible to allow for country and health-system-specific modifications.

## Implementing evidence-based PA promotion in primary healthcare practice—addressing the research to policy/practice gap

Despite observational studies showing that a range of factors influence the extent to which PA promotion interventions are implemented in primary healthcare [21] and implementation science frameworks that propose a multi-factor approach to implementation [20, 22], existing interventions to improve implementation have predominantly focused on individual health practitioner-level strategies such as educational outreach [23]. These strategies have achieved limited success, likely because they fail to address the range of factors that influence implementation, such as supportive policy and integration with existing health system infrastructure [23].

The Practical, Robust Implementation and Sustainability Model (PRISM) [22], which incorporates the Reach, Effectiveness, Adoption, Implementation and Maintenance (RE-AIM) evaluation framework, [24] can be used to guide policy and strategies to support the adoption and implementation of PA promotion in primary healthcare, and evaluate the implementation and effectiveness outcomes. The PRISM has been used in several studies that examine implementation of innovations [25, 26]. For example, the model was used in the United States (U.S.) Veterans Health Administration, which is the largest integrated health care system in the U.S. [25]. It was applied to assess contextual factors throughout intervention planning, implementation, evaluation, and dissemination across health services [25]. The study found that PRISM framework was useful in examining issues related to implementation across different interventions [25]. Another study integrated the PRISM with best practices in Clinical Decision Support Design [26]. Findings from a randomised controlled trial demonstrated positive effects of the PRISM/Clinical Decision Support best practice approach on prescribing for heart failure in primary health care [26].

The PRISM focuses on addressing factors at multiple levels and suggests that several elements are associated with the success of the implementation and sustainability of interventions, including factors relating to the external environment (e.g. international and national policies), recipients and stakeholders, health system infrastructure, and the intervention itself [23]. Evaluation is important to understand the effectiveness of implementation and to inform future interventions, given the lack of evidence to guide the implementation of PA promotion in primary healthcare.

**Fig. 1:**
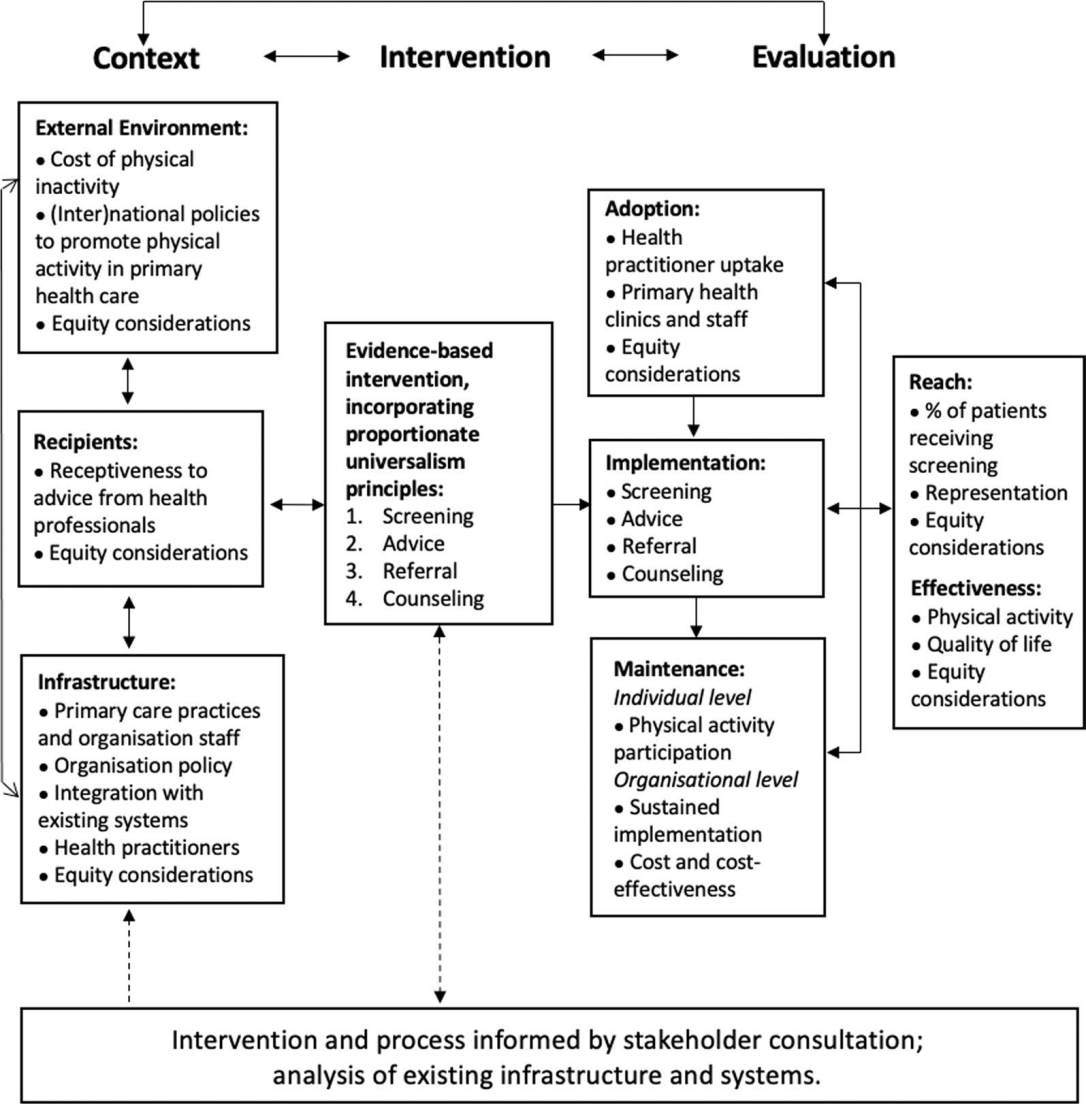
*ProPRISM*; Proportionate (Pro) universalism principles blended with PRISM, adapted from Feldstein and Glasgow (2018)

We revised and assessed evidence-based strategies and interventions from PA promotion research and practice and from other areas of health services research and practice and incorporated them into the *ProPRISM* model. We adapted the PRISM and blended it with the principles of proportionate universalism to support the implementation of PA advice, referral, and counselling in primary healthcare in a way that can help to address health inequities and inform policy. The blended model, based on the proportionate universalism (Pro) principles and PRISM called ‘ProPRISM’, is available in Fig. 1. We suggest future studies to test this model and provide evidence of its effectiveness.

## Application of the *ProPRISM*—an Australian case study

In this section, we address each of the ‘Context’ factors in the *ProPRISM* that relate to the implementation of evidence-based PA promotion in primary healthcare. Using the model, we identify ‘supportive’ factors already in place (e.g., international policies and patient support for PA promotion in primary healthcare) to support evidence-based PA promotion in primary healthcare and make recommendations for factors that are not currently supportive or absent (e.g., policy changes and strategies). We contend that, if these supportive factors are addressed, the evidence-based model of PA promotion in primary healthcare would be adopted, implemented and sustained in practice and consequently it would reach a large proportion of the population and improve PA and quality of life, while addressing health inequities. Due to scarcity of evidence to support the implementation of PA promotion in primary healthcare settings specifically, some evidence-based strategies were adopted from other areas of health service research and practice and therefore need to be tested in the context of PA promotion in primary healthcare.

### External environment

- *Cost of physical inactivity:* The costs of physical inactivity are high. Physical inactivity has been identified as a significant economic burden, with conservative estimates showing annual costs of $805 million, including $640 million in direct healthcare costs [2].
- *(Inter)national policies to promote PA in primary healthcare*: Current national and international policies broadly support PA promotion in health care [27, 28]. Clinical guidelines recommend primary healthcare providers promote PA to their patients via screening, advice, and referral to appropriately qualified practitioners for PA counselling [29]. Australia has a Government Funded exercise referral scheme. Introduced in 2006, the Australian government introduced Chronic Disease Management Plans [30] that enable general practitioners (GPs) to organise and manage multidisciplinary health care for patients with chronic diseases/conditions (e.g. cardiovascular disease, musculoskeletal conditions, cancer, stroke, and diabetes) [7]. Patients can be referred to allied health professionals, including Accredited Exercise Physiologists (AEPs). They can claim a rebate for a maximum of five appointments per calendar year, which can be shared across different allied health professionals or provided by a single allied health professional [7, 30]. Although this policy is promising in terms of recognising the importance of integrating PA into primary healthcare, it is not consistent with best available evidence or clinical guidelines. The current policy does not support routine screening of PA for all patients and only patients with existing chronic conditions are eligible. Further, the policy has not been widely taken up by GPs [10]. An analysis of Medicare Benefits Schedule data showed that only 242,690 AEP services were accessed Australia-wide in that year, representing 0.06% of total Medicare Benefits Schedule services (N = 384,043,993) [7]. Therefore, we recommend that this policy be amended to support evidence-based model of routine screening, advice, and referral for PA counselling for all patients who are insufficiently active.
- *Equity considerations: (Inter)national policies to reduce health inequities*: An equity-based approach is embedded in the WHO *Global Action Plan for the Prevention and Control of Noncommunicable Diseases*. [31] The WHO *Global Action Plan on Physical Activity* recommends the implementation of the action plan be guided by the principle of proportionate universalism [1].* Australia’s long term national health plan* advocates for a more equitable health system, [32] and Australian *National Women’s Health Strategy 2020–2030* [33] and *Men’s Health Strategy 2020–2030* [34] mention reduction of health inequities as one of their key priorities. However, these national policies do not state *how* they will address health inequities. We recommend that policies should put more emphasis on the reduction of health inequities and support Australians living in the most disadvantaged areas (bottom two quintiles, as determined by Socio-Economic Indexes for Areas) to receive more and longer counselling sessions.

### Recipients

- *Receptiveness to advice from health care professionals*: Health care practitioners are considered among some of the most trusted members of society. [35] They often have an important role in influencing positive behaviour change [35] and are considered a trusted source of lifestyle-related information and advice [36]. Health practitioners, especially GPs, establish ongoing relationships and trust with their patients, so their impact on building a more active population can be significant. [35]
- *Equity considerations:* Evidence shows that PA promotion in primary healthcare is effective across various groups. [7] Research demonstrates that PA promotion in primary healthcare is acceptable to adults living in areas of socioeconomic disadvantage. [7]

### Infrastructure

#### Primary healthcare practices and organisation staff:

- Several strategies might be used to support PA promotion in health care organisations, including*:* (1) *A ‘practice facilitator’*: Research shows practice facilitators can improve the quality of health care delivery [37]; (2) *A ‘change champion’ or ‘collective impact facilitator’:* There is substantial evidence from multiple disciplines that change champions or collective impact facilitators can play an important role in implementing innovations. Their value is especially apparent in health care settings [38]; (3) *PA counsellors co-located with GPs*: Face-to-face introductions and building relationships have been shown to encourage GP referrals to allied health practitioners [39]. Evidence suggests that co-location (mono-disciplinary and multi-disciplinary) is associated with positive outcomes at the GP level [40]; (4) *Audit and feedback*: A regular summary of performance is one of the most widely applied interventions for quality improvement in medical practice [41]. It is considered to be effective and important for practitioner improvements and shown to be more successful if provided on a regular basis by a colleague or a supervisor and if it includes a specific action plan or guide [41].

#### Organisation policy:

- *Development of implementation guides:* Well-designed implementation or action plans are particularly important for implementation of interventions within healthcare settings and should be supported [42].

#### Integration within existing systems:

- *Digitisation to enable automated screening and advice prompts for physical inactivity:* Automated screening for PA and other lifestyle and behavioural risk factors have been shown to be feasible for implementation in primary healthcare [43]. This aligns with the rapidly growing practice of digital health to support, promote, and monitor PA to improve overall health and well-being of patients [44]. In terms of prompts for GPs to provide brief advice and refer patients to PA support, some studies have found that reminders such as computerized prompts or chart stickers improved counselling rates for PA [45] .
- *Digital health and telephone triage*: In hard to reach, rural and remote locations health practitioners could provide telephone advice and counselling and utilise digital health tools. To increase the reach and uptake of PA advice and counselling by people living in rural and remote areas, phone advice may be a valuable substitute when face-to-face advice is hard to achieve. This could significantly improve the accessibility of services. Research shows no significant difference in patient satisfaction using telephone triage for counselling services compared to other forms of care [46]. Moreover, digital health tools can support accessibility, engagement, and personalised advice in disadvantaged areas [47]. Evidence suggests phone and digital delivery of PA advice and counselling are effective in the general population [11] and in socioeconomically disadvantaged population groups [48].

#### Health practitioners:

- *PA education for medical students*: Research shows that medical students lack knowledge on benefits of PA and PA recommendations [49, 50]. Although the majority of Australian medical schools report some training related to PA, guidance on PA recommendations is not routinely provided [49]. Improvement of PA education for medical students may result in more medical practitioners valuing PA promotion and adopting this in their practice [50].
- *Upskilling generalist allied health professionals to provide PA counselling:* In some areas, there is a significant shortage of AEP’s, so socio-economically disadvantaged groups and people living in rural and remote regions have limited access to PA counsellors. Evidence suggests that PA counselling can be effectively delivered by health professionals from a range of backgrounds [13]. Health professionals such as psychologists, social workers, and/or occupational therapists could provide counselling services for people without complex conditions. These health professionals would need to be knowledgeable about local PA resources, undertake training in PA behaviour change counselling, and get accreditation for providing PA counselling. However, it should be noted that multiple factors influence the practices of health practitioners (e.g. education, knowledge, awareness, attitudes, and self-efficacy), which can be barriers to, or facilitators of, their behaviour change and willingness to upskill and provide PA counselling.

#### Equity considerations:

- *Encouraging practitioners to work in areas of disadvantage:* Recruiting exercise practitioners and other health practitioners to disadvantaged areas is an ongoing challenge [51]. Research shows that recruiting and training students from rural and remote backgrounds to become health practitioners may increase the distribution of health practitioners to areas of disadvantage [52]. Furthermore, rurally-orientated medical education programs such as rural clinical placements and rurally relevant curricula may impact students’ decision to practice in these areas [51].
- *Higher rebates for AEPs in disadvantaged areas*: Although the need for services is higher in areas of greater disadvantage, fewer AEPs are practising in disadvantaged areas and those who do have higher caseloads. Moreover, low consultation fees that characterise service fees for AEPs in disadvantaged areas is likely to make practice unsustainable in these areas [53] and encourage practitioners to locate in more affluent areas where people have more means to provide co-payments for services [7]. AEPs who work in areas of greatest disadvantage should receive higher rebates. This would: (1) attract more AEPs to work in low-income areas and would thus increase the availability of services to match the higher need in disadvantaged areas, and (2) result in longer consultation times to manage the complex needs of disadvantaged patients, which will likely result in better health outcomes and cost savings. One of the key negative factors influencing health practitioners’ level of satisfaction with working conditions in disadvantaged communities was “suboptimal remuneration” [52] which indicates that higher financial incentives and rebates might contribute to attracting and retaining practitioners in these areas.

## Conclusion

Given the wide population reach of primary healthcare, implementation and scale-up of evidence-based PA promotion interventions has the potential to increase population levels of PA and to reduce rates of chronic disease and health care costs. In this commentary, we proposed a novel model, adapted from implementation science, to support the delivery and implementation of PA promotion in primary healthcare and to inform policy and practice. The model shows that there is no ‘quick fix’ to increasing the implementation of PA in primary healthcare and a systems approach supported by evidence-based policies and strategies is needed for lasting change. In the *ProPRSIM*, we blended the principles of proportionate universalism and applied an equity lens to help to address the lower levels of PA among socio-economically disadvantaged groups and thus reduce health inequities. Even though we used Australia as a case study, the model can be adjusted and applied to other high-income countries. Future studies should test the model and provide evidence of its effectiveness and cost-effectiveness when delivered in routine practice.

There is potential to expand the proposed model to other health sectors (e.g., secondary and tertiary care). Furthermore, this approach could also be translated to address other chronic disease risk factors such as diet, smoking, and alcohol consumption. Thus, this approach has the potential to address multiple modifiable risk factors and transform the delivery of health care to a prevention-focused health service model. This could reduce the prevalence and burden of chronic disease and health care costs in high-income countries.

## Data Availability

Not applicable.

## Abbreviations

AEP: Accredited exercise physiologist
GP: General practitioner
PA: Physical activity
PRISM: Practical, robust implementation and sustainability model
WHO: World Health Organization
